# Ancient Mitogenomes Reveal the Maternal Genetic History of East Asian Dogs

**DOI:** 10.1093/molbev/msae062

**Published:** 2024-03-20

**Authors:** Ming Zhang, Yanbo Song, Caihui Wang, Guoping Sun, Lina Zhuang, Mingjian Guo, Lele Ren, Shargan Wangdue, Guanghui Dong, Qingyan Dai, Peng Cao, Ruowei Yang, Feng Liu, Xiaotian Feng, E Andrew Bennett, Xiaoling Zhang, Xi Chen, Fen Wang, Fengshi Luan, Wenbin Dong, Guoquan Lu, Daohua Hao, Hongwei Hou, Hui Wang, Hong Qiao, Zhongxin Wang, Xiaojun Hu, Wei He, Lin Xi, Weilin Wang, Jing Shao, Zhouyong Sun, Lianjian Yue, Yan Ding, Norbu Tashi, Yang Tsho, Yan Tong, Yangheshan Yang, Shilun Zhu, Bo Miao, Wenjun Wang, Lizhao Zhang, Songmei Hu, Xijun Ni, Qiaomei Fu

**Affiliations:** China-Central Asia “the Belt and Road” Joint Laboratory on Human and Environment Research, Key Laboratory of Cultural Heritage Research and Conservation, School of Culture Heritage, Northwest University, Xi’an, China; Key Laboratory of Vertebrate Evolution and Human Origins, Institute of Vertebrate Paleontology and Paleoanthropology, Center for Excellence in Life and Paleoenvironment, Chinese Academy of Sciences, Beijing, China; School of Archaeology, Shandong University, Jinan, China; China-Central Asia “the Belt and Road” Joint Laboratory on Human and Environment Research, Key Laboratory of Cultural Heritage Research and Conservation, School of Culture Heritage, Northwest University, Xi’an, China; Zhejiang Provincial Institute of Cultural Relics and Archaeology, Hangzhou, China; National Museum of China, Beijing, China; National Museum of China, Beijing, China; School of History and Culture, Lanzhou University, Lanzhou, China; Tibet Institute for Conservation and Research of Cultural Relics, Lhasa, China; Key Laboratory of Western China's Environmental Systems (Ministry of Education), College of Earth and Environmental Sciences, Lanzhou University, Lanzhou, China; Key Laboratory of Vertebrate Evolution and Human Origins, Institute of Vertebrate Paleontology and Paleoanthropology, Center for Excellence in Life and Paleoenvironment, Chinese Academy of Sciences, Beijing, China; Key Laboratory of Vertebrate Evolution and Human Origins, Institute of Vertebrate Paleontology and Paleoanthropology, Center for Excellence in Life and Paleoenvironment, Chinese Academy of Sciences, Beijing, China; Key Laboratory of Vertebrate Evolution and Human Origins, Institute of Vertebrate Paleontology and Paleoanthropology, Center for Excellence in Life and Paleoenvironment, Chinese Academy of Sciences, Beijing, China; Key Laboratory of Vertebrate Evolution and Human Origins, Institute of Vertebrate Paleontology and Paleoanthropology, Center for Excellence in Life and Paleoenvironment, Chinese Academy of Sciences, Beijing, China; Key Laboratory of Vertebrate Evolution and Human Origins, Institute of Vertebrate Paleontology and Paleoanthropology, Center for Excellence in Life and Paleoenvironment, Chinese Academy of Sciences, Beijing, China; Key Laboratory of Vertebrate Evolution and Human Origins, Institute of Vertebrate Paleontology and Paleoanthropology, Center for Excellence in Life and Paleoenvironment, Chinese Academy of Sciences, Beijing, China; Key Laboratory of Vertebrate Evolution and Human Origins, Institute of Vertebrate Paleontology and Paleoanthropology, Center for Excellence in Life and Paleoenvironment, Chinese Academy of Sciences, Beijing, China; Department of Cultural Heritage and Museology, Nanjing Normal University, Nanjing, China; School of Archaeology, Shandong University, Jinan, China; School of Archaeology, Shandong University, Jinan, China; Shandong Provincial Institute of Cultural Relics and Archaeology, Jinan, China; School of Archaeology, Shandong University, Jinan, China; Shandong Provincial Institute of Cultural Relics and Archaeology, Jinan, China; Gansu Provincial Institute of Cultural Relics and Archaeology, Lanzhou, China; Gansu Provincial Institute of Cultural Relics and Archaeology, Lanzhou, China; Fudan Archaeological Science Institute, Fudan University, Shanghai, China; Qinghai Provincial Cultural Relics and Archaeology Institute, Xining, China; Qinghai Provincial Cultural Relics and Archaeology Institute, Xining, China; Qinghai Provincial Cultural Relics and Archaeology Institute, Xining, China; Tibet Institute for Conservation and Research of Cultural Relics, Lhasa, China; Shaanxi Academy of Archaeology, Xi’an, China; School of Archaeology and Museology, Shanxi University, Taiyuan, China; Shaanxi Academy of Archaeology, Xi’an, China; Shaanxi Academy of Archaeology, Xi’an, China; Shaanxi Academy of Archaeology, Xi’an, China; Shaanxi Academy of Archaeology, Xi’an, China; Tibet Institute for Conservation and Research of Cultural Relics, Lhasa, China; Tibet Institute for Conservation and Research of Cultural Relics, Lhasa, China; Tibet Institute for Conservation and Research of Cultural Relics, Lhasa, China; School of Ecological and Environmental Sciences, East China Normal University, Shanghai, China; Key Laboratory of Vertebrate Evolution and Human Origins, Institute of Vertebrate Paleontology and Paleoanthropology, Center for Excellence in Life and Paleoenvironment, Chinese Academy of Sciences, Beijing, China; University of the Chinese Academy of Sciences, Beijing, China; Key Laboratory of Vertebrate Evolution and Human Origins, Institute of Vertebrate Paleontology and Paleoanthropology, Center for Excellence in Life and Paleoenvironment, Chinese Academy of Sciences, Beijing, China; Key Laboratory of Vertebrate Evolution and Human Origins, Institute of Vertebrate Paleontology and Paleoanthropology, Center for Excellence in Life and Paleoenvironment, Chinese Academy of Sciences, Beijing, China; Science and Technology Archaeology, National Centre for Archaeology, Beijing, China; Key Laboratory of Vertebrate Evolution and Human Origins, Institute of Vertebrate Paleontology and Paleoanthropology, Center for Excellence in Life and Paleoenvironment, Chinese Academy of Sciences, Beijing, China; Joint International Research Laboratory of Environmental and Social Archaeology, Shandong University, Qingdao, China; Shaanxi Academy of Archaeology, Xi’an, China; Key Laboratory of Vertebrate Evolution and Human Origins, Institute of Vertebrate Paleontology and Paleoanthropology, Center for Excellence in Life and Paleoenvironment, Chinese Academy of Sciences, Beijing, China; University of the Chinese Academy of Sciences, Beijing, China; Key Laboratory of Vertebrate Evolution and Human Origins, Institute of Vertebrate Paleontology and Paleoanthropology, Center for Excellence in Life and Paleoenvironment, Chinese Academy of Sciences, Beijing, China; University of the Chinese Academy of Sciences, Beijing, China

**Keywords:** ancient DNA, mitochondrial DNA, genetic history, east Asia, *Canis lupus familiaris*

## Abstract

Recent studies have suggested that dogs were domesticated during the Last Glacial Maximum (LGM) in Siberia, which contrasts with previous proposed domestication centers (e.g. Europe, the Middle East, and East Asia). Ancient DNA provides a powerful resource for the study of mammalian evolution and has been widely used to understand the genetic history of domestic animals. To understand the maternal genetic history of East Asian dogs, we have made a complete mitogenome dataset of 120 East Asian canids from 38 archaeological sites, including 102 newly sequenced from 12.9 to 1 ka BP (1,000 years before present). The majority (112/119, 94.12%) belonged to haplogroup A, and half of these (55/112, 49.11%) belonged to sub-haplogroup A1b. Most existing mitochondrial haplogroups were present in ancient East Asian dogs. However, mitochondrial lineages in ancient northern dogs (northeastern Eurasia and northern East Asia) were deeper and older than those in southern East Asian dogs. Results suggests that East Asian dogs originated from northeastern Eurasian populations after the LGM, dispersing in two possible directions after domestication. Western Eurasian (Europe and the Middle East) dog maternal ancestries genetically influenced East Asian dogs from approximately 4 ka BP, dramatically increasing after 3 ka BP, and afterwards largely replaced most primary maternal lineages in northern East Asia. Additionally, at least three major mitogenome sub-haplogroups of haplogroup A (A1a, A1b, and A3) reveal at least two major dispersal waves onto the Qinghai-Tibet Plateau in ancient times, indicating eastern (A1b and A3) and western (A1a) Eurasian origins.

## Introduction

The gray wolf (*Canis lupus*) was the first species to be domesticated, eventually giving rise to dogs. Dogs have accompanied humans on every continent they have inhabited as demonstrated by archaeological records (Frantz et al. 2016; Bergström et al. 2020) and genetic studies (Pang et al. 2009; Thalmann et al. 2013; Peng et al. 2015; Song et al. 2016; Ní-Leathlobhair et al. 2018; Ameen et al. 2019; Bergström et al. 2020; Zhang et al. 2020; Perri et al. 2021); thus, the genetic history of dogs also reflects human history (Frantz et al. 2016; Ní-Leathlobhair et al. 2018; Bergström et al. 2020; Zhang et al. 2020; Perri et al. 2021). Despite their importance in human history, the timing of their domestication and geographical origins are still unclear. Potential domestic centers have been proposed, including Europe (Thalmann et al. 2013), the Middle East (VonHoldt et al. 2010), and southern East Asia (Pang et al. 2009; Wang et al. 2016). Genomic data have shown that southern East Asian gray wolves (the likely ancestor of dogs based on modern genetic studies) originate from a single lineage and may form a distinct sub-population (Wang et al. 2019) suggested the dogs may not originate from southern East Asia. Ancient dog remains from the northern part of East Asia are older and more numerous than in the southern part (Wu 2014; Zhao 2014; Ren and Dong 2016), indicating that dogs at least appeared in northern East Asia before they are recorded in the south, contradicting the expectations of a southern East Asian origin hypothesis.

Recent studies based on ancient DNA (aDNA) have suggested that dogs may have originated in Siberia (Perri et al. 2021). Dogs are generally more closely related to ancient wolves from eastern Eurasia (northern East Asia and eastern Siberia) than to those from western Eurasia (Europe and the Middle East), and an eastern Eurasian-related source appears to have contributed approximately 100% of the ancestry of early dogs in Siberia, the Americas, East Asia, and northeastern Europe (Bergström et al. 2022). Perri et al. (2021) proposed that during the harsh environment of the last glacial maximum (LGM), the ancestor of dogs was domesticated due to coexistence with humans in Siberia, also suggesting a domestication process in eastern Eurasia. These studies have shown that aDNA can be used to understand dog evolution in a powerful way. More aDNA data from East Asia will improve our understanding of the genetic origins of dogs.

To establish a more precise geographic and temporal framework for dog genetic history in East Asia, we recently sequenced 102 complete mitochondrial genomes of ancient individuals (see supplementary table S1, Supplementary Material online). Published ancient and modern mitogenomes (see supplementary table S2, Supplementary Material online) were also selected to profile the maternal genetic history and migration waves of dogs in East Asia.

## Results

### Sample Information

**Fig. 1.**
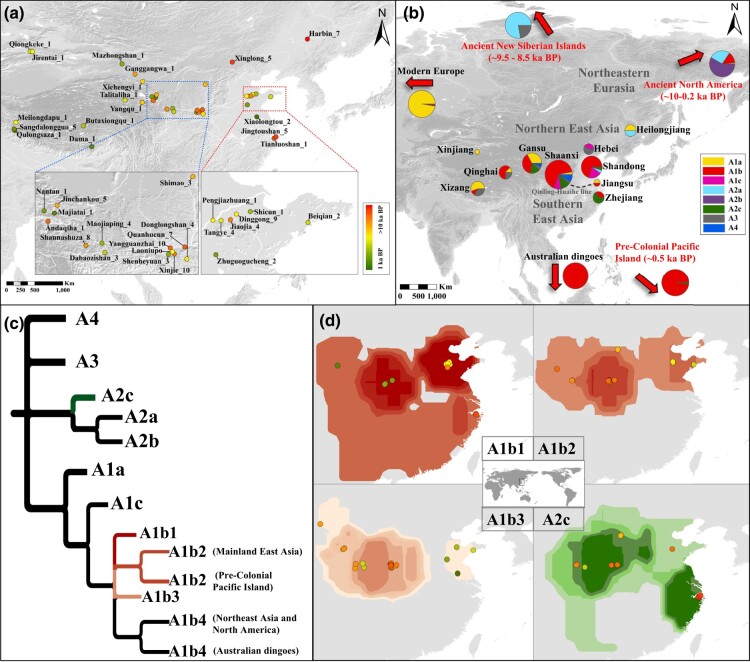
a) Geographic locations of different archaeological sites. The dots depict all 38 sites from East Asia, where the number of specimens is listed after the location. The samples are marked with colors ranging from red to green, indicating the range of their ages from older to younger. Two concentrated areas are enlarged for better viewing. Base map downloaded from Natural Earth (www.naturalearthdata.com). b) A map of China with pie charts showing the location of the specimens and associated sub-haplogroups belonging to haplogroup A. Data of modern Europe, Australian dingoes, precolonial Pacific Island, and ancient Siberia were cited from Zhang et al. (2020); and North America was cited from Ameen et al. (2019). c) A simplified tree was based on a Bayesian phylogenetic tree (see supplementary fig. S3, Supplementary Material online). Four main East Asian sub-haplogroups (A1b1, A1b2, A1b3, and A2c) are marked in different colors. d) Geographic distribution heatmaps of four main East Asian sub-haplogroups (A1b1, A1b2, A1b3, and A2c). The dots depict ancient individuals of each sub-haplogroups, and colors represent the age of the samples.

In this study, we used aDNA capture techniques (Fu et al. 2013) and successfully obtained 102 complete mitogenomes (depths of 5.3- to 843.1-fold coverage, mean 102.9-fold) from 12.9 to 1 ka BP (1,000 years before present) dog specimens from East Asia (Fig. 1a; see supplementary table S1, Supplementary Material online). A total of 120 ancient East Asian individuals (119 dogs and one wolf) were used after combining with the individuals from Zhang et al. (2020) spanning from the Palaeolithic to the historical periods. One individual from Harbin, Heilongjiang, identified by its mitogenome as a dog, was radiocarbon dated to 12.9 ka Cal BP, thus representing the oldest convincing dog remains found in East Asia. We also sequenced individuals older than 8 ka BP from the Xinglong site, Hebei Province (Fig. 1a; see supplementary table S1, Supplementary Material online). Four individuals from Harbin were estimated by tip-dating method of BEAST v1.10.4 to range from between 16.1 and 6.3 ka BP (see supplementary table S4 and fig. S6, Supplementary Material online). A high-quality sequence belonging to haplogroup A1a was estimated to be about 10.2 ka BP (95% highest posterior density [HPD] interval, 13.5 to 6.8 ka BP) and represented the deepest lineage of haplogroup A1a (see supplementary table S4, Supplementary Material online and supplementary fig. S6, Supplementary Material online). Although rarely found, dog remains in southern East Asia play an important role in resolving dog domestication history based on modern genetics and archaeological records (see supplementary fig. S1, Supplementary Material online). To this end, we also recovered mitogenome sequences from the oldest dog remains found south of the Yangtze River (8.3 ka BP, from the Jingtoushan site, Zhejiang province). Selected ancient and modern dogs for comparison are showed in supplementary table S2, Supplementary Material online.

### Haplogroup Nomenclature System

**Fig. 2.**
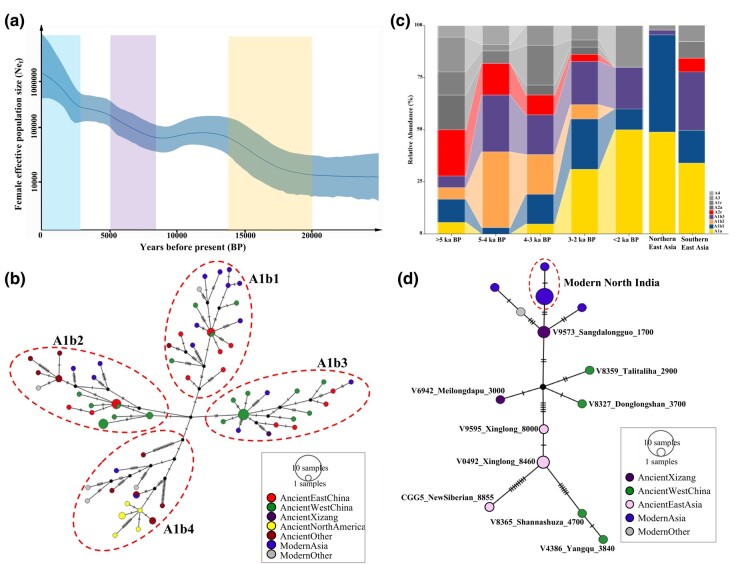
a) Bayesian skyline plot based on 188 individuals for effective female population size (Ne_f_) estimation. The plot shows three obvious expansions: the first one starts at approximately 20 to 19 ka BP, the second one starts at approximately 8 to 7.5 ka BP, and the third one starts at approximately 3 to 2.5 ka BP. b) Median-joining network of sub-haplogroup A1b, divided into four main lineages (A1b1, A1b2, A1b3, and A1b4). Haplotypes are represented by circles whose sizes are proportional to the number of individuals. c) Haplogroup proportion of different time periods (five time periods spanning the Neolithic to the Iron Age) in East Asia. Ancient individuals within sub-haplogroup A1a found in East Asia increased quickly after 3 ka BP. d) Median-joining network of sub-haplogroup A3. It showed clear connections among ancient dogs from northeastern Eurasia (New Siberian Islands, approximately 9 ka BP), northern China (Xinglong, approximately 8.5 to 8 ka BP), western China (Donglongshan, approximately 3.7 ka BP), and the Qinghai-Tibet Plateau (Talitaliha, approximately 2.9 ka BP; Meilongdapu, approximately 3 ka BP; and Sangdalongguo, approximately 1.7 ka BP).

Ancient East Asian dogs in this study belonged to haplogroups A (112/119, 94.12%), B (4/119, 3.36%), and C (3/119, 2.52%) (see supplementary figs. S2 to S4, tables S1 and S3, Supplementary Material online), other haplogroups (e.g. D, E, and F) do not appear in our dataset. Among haplogroup A, half of the ancient dogs in this study belonged to sub-haplogroup A1b (55/112, 49.11%), and other major sub-haplogroups included A1a (18/112, 16.07%), A2c (12/114, 10.71%), A3 (10/112, 8.93%), A1c (9/112, 8.04%), A2a (4/112, 3.57%), and A4 (4/112, 3.57%) (see supplementary figs. S3, S4 and table S3, Supplementary Material online). Sub-haplogroups A1b, A1c, and A3 were widely distributed throughout East Asia, and were typical sub-haplogroups of this region (Fig. 1b to d; see supplementary figs. S3, S4 and table S3, Supplementary Material online). Sub-haplogroup A1a was concentrated in the western Eurasia and is a typical western Eurasia sub-haplogroup (Figs. 1b and  2c; see supplementary figs. S3, S4 and table S3, Supplementary Material online).

### Haplogroup Comparison and Network Construction Results

**Fig. 3.**
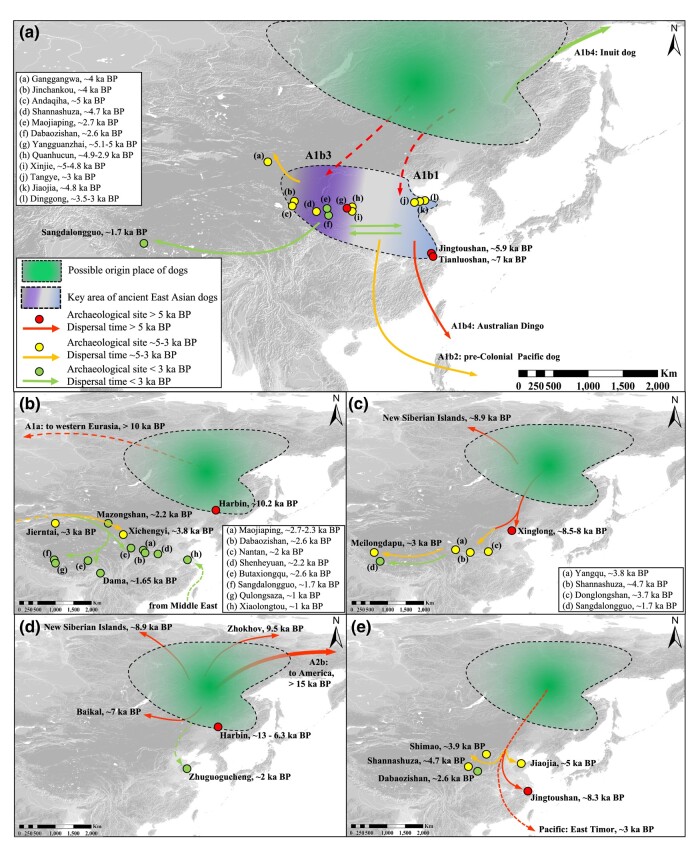
Main migratory events associated with the maternal genetic history of dogs from the Neolithic to Iron Age (approximately 12.9 to 1 ka BP), derived from a synthesis of available evidence. The specific migratory events of dogs belonging to haplogroup A1b as in (a); A1a as in (b); A3 as in (c); A2 as in (d), and A2c as in (e).

The median-joining network of haplogroup A showed a clear division of each sub-haplogroup (A1a, A1b1, A1b2, A1b3, A1b4, A1c, A2a, A2b, A2c, A3, and A4) with a star-like structure (see supplementary fig. S5A, Supplementary Material online). The haplogroup comparison and network construction results of important sub-haplogroups were as follows:

1. A1a (see supplementary fig. S5B, Supplementary Material online): The deepest lineage of sub-haplogroup A1a was represented by one individual found in Harbin, Heilongjiang (estimated by BEAST tip dating approximately 10.2 ka BP) (see supplementary fig. S6 and table S4, Supplementary Material online). This lineage split from other sub-haplogroup A1a lineages approximately 15 ka BP (see supplementary fig. S3, Supplementary Material online), and the most recent common ancestor (TMRCA) of all other modern and ancient western Eurasian sub-haplogroup A1a individuals is estimated to date back to approximately 10 ka BP (see supplementary fig. S3, Supplementary Material online). Apart from the deepest Harbin lineage, the oldest individual of sub-haplogroup A1a dates to approximately 7 ka BP and was found in the Middle East (Fig. 3b; see supplementary table S2, Supplementary Material online). The network of sub-haplogroup A1a shows a single center and multiple branches (see supplementary fig. S5B, Supplementary Material online), indicating that sub-haplogroup A1a derived multiple new haplotypes in a short time, consistent with a rapid population expansion event (Fig. 2a). In general, sub-haplogroup A1a predominates among dogs in western Eurasia and almost all the modern European breed dogs belong to this sub-haplogroup (Zhang et al. 2020). The maternal lineages of later East Asian dogs (<4 ka BP) belonging to this sub-haplogroup apparently originated from western Eurasian dogs (see supplementary figs. S3, S4 and S5B, Supplementary Material online). The Tibetan lineage, with ancient individuals from Xinjiang, Gansu, and Shaanxi, marked in dashed red lines in supplementary fig. S5B, Supplementary Material online, clearly shows the dispersal of sub-haplogroup A1a dogs from western China to the Qinghai-Tibet Plateau.
2. A1b (Fig. 2b): Sub-haplogroup A1b is a main lineage of ancient dogs in East Asia (55/119 = 46.22%), particularly individuals older than 2.7 ka BP from the Yangtze and Yellow River Basins (43/70 = 61.4%) (Fig. 1d; see supplementary table S1, Supplementary Material online). The younger individual from Jingtoushan (approximately 5.9 ka BP) belongs to sub-haplogroup A1b and is basal to all other A1b lineages (A1b1, A1b2, A1b3, and A1b4), suggesting that it might originate from the ancestral population of sub-haplogroup A1b. Ancient northern East Asian dogs are grouped into two major groups. One is the “Ancient Western China” (AWC: Shaanxi, Gansu, Qinghai, and Xizang), which was characterized by sub-haplogroup A1b3 (Shaanxi, *n* = 9; Gansu, *n* = 5; Qinghai, *n* = 3; and Xizang, *n* = 1) and sub-haplogroup A3 (Xizang, *n* = 3; Qinghai, *n* = 2; Shaanxi, *n* = 1; and Gansu, *n* = 1). The other is the “Ancient Eastern China” (AEC: Shandong and Zhejiang), which was characterized by sub-haplogroup A1b1 (Shandong, *n* = 7; and Zhejiang, *n* = 1) (Fig. 2b; see supplementary figs. S3, S4 and table S1, Supplementary Material online). Eighteen individuals from the AWC belonged to sub-haplogroup A1b3 (5.1 to 1.7 ka BP); however, a few younger individuals from the AEC (*n* = 4, 2.7 to 1 ka BP) (see supplementary table S1, Supplementary Masterial online) were significantly derived from sub-haplogroup A1b3 of the AWC (Fig. 2b; see supplementary figs. S3 and S4, Supplementary Material online), indicating eastward gene flow from the AWC to the AEC groups after the establishment of these two dog populations (Figs. 2b and 3a). Similarly, the AEC also contributed to the gene pool for the AWC based on the appearance of younger individuals of sub-haplogroup A1b1 (after 2.2 ka BP) and apparently derived from older individuals of the AEC (4.7 to 3 ka BP) (Figs. 2b and 3a; see supplementary figs. S3 and S4, Supplementary Material online).
3. A2 (see supplementary fig. S5C, Supplementary Material online): Sequences of sub-haplogroup A2 could be further divided into three main sub-haplogroups (A2a, A2b, and A2c). Sub-haplogroups A2a and A2b consist mainly of individuals from ancient North America and ancient East Asia. The oldest dog sample from East Asia (the approximately 12.9 ka BP individual from Harbin, Heilongjiang) belonged to sub-haplogroup A2 (see supplementary figs. S3, S4 and table S1, Supplementary Material online) and was basal to all lineages of sub-haplogroups A2a and A2b. This individual may originate from the common ancestral population of sub-haplogroups A2a and A2b supporting the connection between populations from North America and Northeast Eurasia (Fig. 3d). Two younger individuals from Harbin, Heilongjiang and one from Zhuguogucheng, Shandong, China (approximately 2 ka BP) are also in this sub-haplogroup (see supplementary fig. S5C and table S1, Supplementary Material online). Sub-haplogroup A2c is a typical lineage of East Asia. The basal lineage (represented by one individual from East Timor, approximately 3 ka BP) of sub-haplogroup A2c separated from others approximately 15 ka BP (supplementary fig. S3, Supplementary Material online). However, the 12.9 ka BP northeastern Eurasian individual (Harbin, Heilongjiang) at the base of the A2c lineages (supplementary fig. S5C, Supplementary Material online), indicates that the East Timor individual was also derived from northeastern Eurasian population. All earlier individuals from the Jingtoushan site (approximately 8.3 to 8.1 ka BP) belonged to sub-haplogroup A2c (see supplementary figs. S3, S4 and table S1, Supplementary Material online), which is almost exclusively found in ancient dogs from Shandong (approximately 5 ka BP), Shaanxi (5 to 3.9 ka BP), Gansu (4.7 to 2.6 ka BP), and also appear in a few modern dogs from southern East Asia (Fig. 1b; see supplementary figs. S3, S4 and table S1, Supplementary Material online).
4. A3 (Fig. 2b): Ancient individuals within sub-haplogroup A3 were from Russia: Siberia (New Siberian Islands, approximately 8.8 ka BP) and China: Hebei (Xinglong, approximately 8.5 to 8 ka BP), Gansu (Shannashuza, approximately 4.7 ka BP), Shaanxi (Donglongshan, approximately 3.7 ka BP), Qinghai (Yangqu, approximately 3.8 ka BP and Talitaliha, approximately 2.9 ka BP) and Xizang (Meilongdapu, approximately 3 ka BP and Sangdalongguo, approximately 1.7 ka BP) (see supplementary tables S1 and S2, Supplementary Material online), and modern dogs with sub-haplogroup A3 are from the Qinghai-Tibet Plateau and surrounding area (Fig. 2d; see supplementary table S2, Supplementary Material online). The network structure indicates a clear pattern of derived relationships among populations of sub-haplogroup A3 across different times and geographic locations, suggesting a potential dispersal route onto the Qinghai-Tibet Plateau for this lineage (Figs. 2d and 3c).

### Phylogenetic Trees and Population Demographic Analysis

Both maximum likelihood and Bayesian phylogenetic trees unambiguously showed that all sequences grouping into nine sub-haplogroups that were then renamed according to phylogenetic relationships, as shown in supplementary figs. S3 and S4, Supplementary Material online, and the sequences of haplogroup A were divided into four major sub-haplogroups (A1, A2, A3, and A4) (see supplementary table S3, Supplementary Material online).

A Bayesian skyline plot revealed three distinct expansions in effective female population size (Ne_f_): the first occurring approximately 20 to 19 ka BP; the second approximately 8 to 7.5 ka BP; and the third approximately 3 to 2.5 ka BP (Fig. 2a). Investigation of haplogroup proportions during different periods in East Asia indicated a rapid increase in ancient individuals within sub-haplogroup A1a after 3 ka BP (Fig. 2c).

## Discussion

### Probable Northeastern Eurasian Origin and Southwards Expansion of East Asian Dogs

Recently, Perri et al. (2021) proposed that during the harsh environment of the LGM, the ancestors of dogs were domesticated in Siberia by approximately 23 ka BP. Bergström et al. (2022) by demonstrating that dogs share a closer genetic affinity with ancient wolves from eastern Eurasia rather than those from western Eurasia. This also implies a domestication process centered in eastern Eurasia. After comprehensive examinations of zooarchaeological studies across East Asia, a notable disparity emerges in the distribution of dog remains. These findings indicate a significantly earlier and more abundant presence of dog remains in northern East Asia compared to southern East Asia. The absence of dog remains in southern East Asia before 8.3 ka BP is not likely to be attributable to biases in the archaeological record caused by poor preservation of remains due to the acidic soil or a humid climate of southern East Asia, since older, nondog remains are abundant at multiple southern East Asian archaeological sites (e.g. Wu 2014; Zhao 2014) (see supplementary fig. S1, Supplementary Material online). Moreover, the maternal lineages of ancient dogs from northern East Asia were deeper than the ancient and modern dogs from the south (see supplementary figs. S3, S4 and table S1, Supplementary Material online). The absence of archaeological evidence and the presence of younger maternal lineages suggest a lack of dogs in southern East Asia before 8.3 ka BP (see supplementary fig. S1, Supplementary Material online), further supporting a northeastern Eurasia origin for East Asian domesticated dogs (Perri et al. 2021; Bergström et al. 2022) after the LGM (Fig. 3).

Based on the Bayesian skyline plot and phylogenetic tree (Fig. 2a; see supplementary fig. S3, Supplementary Material online), we showed that the TMRCA of the major sub-haplogroups (A1, A2, A3, and A4) of haplogroup A dates back to approximately 20 ka BP (95% HPD interval, 21.9 to 17.8 ka BP), with most sub-haplogroups appearing shortly after this period, coinciding with a significant increase in the Ne_f_ of dogs (Fig. 2a; see supplementary figs. S3 and S5A, Supplementary Material online). The diversity of mitogenome haplogroups was high in East Asian dog populations, especially ancient dog populations in northern East Asia (see supplementary table S1, Supplementary Material online). Due to the rapidly increasing effective population with a stable natural environment, numerous mitogenome haplogroups emerged soon after the end of the LGM, and East Asian dogs preserved most haplogroups due to the close geographical distance (see supplementary table S1, Supplementary Material online).

Individuals from northern East Asia existed earlier and are more abundant in archaeological records (e.g. Wu 2014; Zhao 2014) (see supplementary fig. S1, Supplementary Material online), and the lineages are deeper than those of ancient and modern dogs from the south (see supplementary figs. S2, S3 and table S1, Supplementary Material online), supporting the southward dispersal of dogs from northern East Asia (Fig. 3). Sub-haplogroup A1b is a main lineage of ancient dogs in East Asia (55/119 = 46.22%), particularly of individuals older than 2.7 ka BP from the Yellow River and Yangtze River Basins (YYRB) (43/70 = 61.4%) (Fig. 1d; see supplementary table S1, Supplementary Material online). The younger individual from Jingtoushan (approximately 5.9 ka BP) belonged to sub-haplogroup A1b and was basal to all other lineages (A1b1, A1b2, A1b3, and A1b4) of sub-haplogroup A1b (see supplementary figs. S3 and S4, Supplementary Material online), indicating that it may originate from the ancestral population of sub-haplogroup A1b, existing around 5.9 ka BP in the lower Yangtze River region. This evidence provided further support to the suggestion that Australian dingoes and Pacific dogs originated in the YYRB (Zhang et al. 2020) (Figs. 1b, 2b, and 3a; see supplementary figs. S3 and S4, Supplementary Material online). The main migratory events of East Asian dogs are also consistent with the southward migratory events of Eastern Eurasians during the Holocene (Zhang and Fu 2020).

The evidence suggests two major possible dispersal directions after dogs were domesticated in northeastern Eurasia. One is southwards to eastern China (e.g. Shandong and Zhejiang) (Fig. 3a). The other is southwestward to western China (e.g. Shaanxi, Gansu, and Qinghai) (Fig. 3a). The maternal genetic difference between the AWC and the AEC gradually formed between 5 and 8 ka BP, and the expansion of the Ne_f_ during this time period was consistent with the development of agricultural civilizations (Fig. 2a) (Zhang et al. 2020, 2022). Some dog populations later spread to southern East Asia, then Southeast Asia, New Guinea, and remote Oceania (Zhang et al. 2020). The results of network analysis also showed that the AWC and the AEC had significantly derived relationships (Figs. 2b and 3a; see supplementary figs. S3 and S4, Supplementary Material online). Combined with the age of these samples, we think this may be due to the interaction between AEC and AWC dog populations, which consisted of increasing east–west exchanges during the Spring and Autumn and Warring States Periods in the first millennium Before Common Era (BCE).

### Several Maternal Lineages of East Asian Dogs Migrating into North America

Studies have demonstrated that dogs in America derive from several expansions from Eurasia (Ní-Leathlobhair et al. 2018; Ameen et al. 2019; Zhang et al. 2020; Perri et al. 2021). Our study confirms that several maternal lineages of ancient dogs (represented by sub-haplogroups A2a, A2b, and A1b4) migrated from an East Asia dispersal into North America.

Sub-haplogroup A2b represented early migrations and is largely present in Siberian dogs and precontact groups in North America, as their remains are found in North America as early as 10 ka BP (Ní-Leathlobhair et al. 2018; Ameen et al. 2019) (see supplementary table S2, Supplementary Material online). While later migrations (after 5.5 ka BP) into the North American Arctic introduced dogs carrying lineages in sub-haplogroups A2a and A1b4 (Fig. 1b) (Ní-Leathlobhair et al. 2018; Ameen et al. 2019; Zhang et al. 2020). However, present populations in North America within sub-haplogroup A1a possibly originate from European dogs (Ní-Leathlobhair et al. 2018; Ameen et al. 2019). In this study, we found that sub-haplogroup A2c commonly found in ancient East Asian dogs clustered with other A2 sub-haplogroups (A2a and A2b), supporting maternal genetic connections between Eurasian and American populations (see supplementary figs. S3 and S4, Supplementary Material online). In particular, an individual from Harbin, Heilongjiang (approximately 12.9 ka BP), was basal to the entire sub-haplogroup A2a and A2b lineages (see supplementary figs. S3, S4 and S5C, Supplementary Material online), indicating that this individual may originate from the common population ancestral to these two sub-haplogroups, and suggesting that the widespread sub-haplogroups (A2a and A2b) in North America are derived from northeastern Eurasian populations (Fig. 3d) (Ní-Leathlobhair et al. 2018; Ameen et al. 2019; Perri et al. 2021). Zhang et al. (2020) proposed that sub-haplogroup A1b4 individuals from western Chukotka (Ní-Leathlobhair et al. 2018) and Alaska to Greenland (Ameen et al. 2019) have a direct connection to ancient East Asian dogs of sub-haplogroup A1b, which suggests that they represent one dog lineage that might have originated from East Asia, arrived in the polar region before 1.75 ka BP, and dispersed further, reaching Greenland before five centuries ago (Fig. 3a) (Ní-Leathlobhair et al. 2018; Ameen et al. 2019; Zhang et al. 2020).

### Western Eurasian Maternal Genetic Component Rapidly Increased in East Asian Dogs After 3 ka BP

Haplogroup C is typical of western Eurasia, as the majority of ancient European dogs (older than 3 ka BP) belonged to haplogroup C (Frantz et al. 2016; Ollivier et al. 2018). Haplogroup C was found in two individuals from Jinchankou, Qinghai (approximately 4 ka BP). Both were close to the mitochondrial sequence of ancient western Eurasian individuals, indicating that the genetic connection between western and eastern Eurasia may date back to at least 4 ka BP (Zhang et al. 2020). Haplogroup B possibly originated from western Eurasian wolves (Thalmann et al. 2013), and individuals of haplogroup B from Xinjiang (Qiongkeke, approximately 2.9 ka BP) and Shandong (Pengjiazhuang, approximately 3.5 ka BP; Tangye, approximately 3 ka BP), also indicated the existence of genetic connections that date back to at least 3.5 ka BP (see supplementary fig. S4 and table S1, Supplementary Material online).

Sub-haplogroup A1a constitutes 62% of haplogroup A lineages found in domesticated dogs worldwide today (Zhang et al. 2020), and almost all sub-haplogroup A1a lineages up to the present day possibly originated from western Eurasia (see supplementary figs. S3, S4 and S5B, Supplementary Material online). Except the deepest lineage (represented by V0722_Harbin), the oldest sub-haplogroup A1a individual of our study was from Xichengyi, Gansu (approximately 3.8 ka BP). This individual also supported the western Eurasian maternal genetic contribution to eastern Eurasian dogs dating back to at least approximately 4 ka BP, which is consistent with the results of haplogroups B and C. However, the frequency of ancient individuals belonging to sub-haplogroup A1a found in East Asia increased quickly after 3 ka BP (Fig. 2c), which is also consistent with mtDNA replacement events found in northern East Asia (Zhang et al. 2020). Combining the appearance of haplogroups (B and C) and sub-haplogroup A1a in East Asian dogs, we believe that mtDNA replacement started in northern East Asia by at least 4 ka BP, and was well underway by 3 ka BP, largely replacing most primary maternal lineages in northern East Asia in the following periods (Fig. 2c). However, southern East Asia and Southeast Asia still retain most of their original maternal lineages (Pang et al. 2009; Peng et al. 2015; Song et al. 2016; Zhang et al. 2020).

One individual from the Xiaolongtou site in Jiangsu (Song Dynasty, approximately 1 ka BP) showed a genetic connection with ancient dogs from the Middle East (see supplementary figs. S3, S4, and S5B, Supplementary Material online). The presence of such a genetic link aligns with the extensive trade activities between East Asia and the Middle East during or before the Song Dynasty (Fig. 3b).

### At Least Two Major Dispersal Waves of Dogs Onto the Qinghai-Tibet Plateau

Dogs migrated onto the Qinghai-Tibet Plateau together with humans. The spread of dogs across the Qinghai-Tibet Plateau in ancient times likely consisted of multiple small-scale migration events but can be delineated into at least two major waves.

The earlier wave onto the Qinghai-Tibet Plateau was represented by individuals within sub-haplogroups A1b and A3. During approximately 5 to 4 ka BP, dogs within sub-haplogroups A1b (A1b2 from Andaqiha, Qinghai, approximately 5 ka BP; Shannashuza, Gansu, approximately 4.7 ka BP; A1b3 from Jinchankou, Qinghai, approximately 4 ka BP) and A3 (from Shannashuza, Gansu, approximately 4.7 ka BP) arrived at the northeast edge of the Qinghai-Tibet Plateau. Together with ancient individuals from Siberia (New Siberian Islands, approximately 8.8 ka BP), Hebei (Xinglong, approximately 8.5 to 8 ka BP), Gansu (Shannashuza, approximately 4.7 ka BP), Shaanxi (Donglongshan, approximately 3.7 ka BP), Qinghai (Yangqu, approximately 3.8 ka BP; Talitaliha, about 2.9 ka BP), and Xizang (Meilongdapu, approximately 3 ka BP; Sangdalongguo, approximately 1.7 ka BP) (see supplementary tables S1 and S2, Supplementary Material online), and modern dogs from the Qinghai-Tibet Plateau and surrounding area (Fig. 2d; see supplementary table S2, Supplementary Material online) (Cai et al. 2023), we can trace the dispersal route of sub-haplogroup A3 populations (Fig. 3c). In summary, dogs belonging to sub-haplogroups A1b and A3 were found to be living at the northeast edge of the Qinghai-Tibet Plateau by at least 5 to 4 ka BP and a small proportion of them arrived onto the Qinghai-Tibet Plateau during this time (Fig. 3c). These maternal lineages have persisted in later dogs from the Qinghai-Tibet Plateau and surrounding area.

The later wave onto the Qinghai-Tibet Plateau is represented by sub-haplogroup A1a found in Xizang (Butaxiongqu, approximately 2.6 ka BP; Sangdalongguo, approximately 1.9 to 1.65 ka BP; Dama, approximately 1.65 ka BP; Qulongsaza, approximately 1 ka BP) and Qinghai (Nantan, approximately 2 ka BP). These individuals indicated the arrival of dogs carrying sub-haplogroup A1a on the Qinghai-Tibet Plateau before 2.6 ka BP (see supplementary figs. S3, S4, S5B and table S1, Supplementary Material online). Together with the ancient individuals of sub-haplogroup A1a found in Xinjiang (Jierentai, approximately 3 ka BP), Gansu (Xichengyi, approximately 3.8 ka BP; Maojiaping, approximately 2.7 to 2.3 ka BP; Dabaozishan, approximately 2.6 ka BP; Mazongshan, approximately 2.2 ka BP), and Shaanxi (Shenheyuan, approximately 2.2 ka BP), they appear to reveal migration routes and timelines from western Eurasia to East Asia before 3.8 ka BP, arriving on the Qinghai-Tibet Plateau before 2.6 ka BP (Fig. 3b; see supplementary fig. S5B, Supplementary Material online). Bergström et al. (2020) suggested that the eastwards migrations of steppe pastoralists had a more substantial impact on dog ancestry than the ancestry of humans in East Asia. Bergström et al. (2020) discuss the spread of steppe-related ancestry throughout East Asia. Our results agree with this proposal and provide a more accurate timeline and details for migration routes.

## Conclusions

East Asia has preserved most dog mitochondrial haplogroups from ancient to modern times, which may attest to its important role in dog domestication. East Asian dogs were likely derived from northeastern Eurasian populations after the LGM (Bergström et al. 2020, 2022; Perri et al. 2021). The dog population began to expand at the end of the LGM (Fig. 2a), coinciding with the warming climate. Two major dispersal directions of domesticated dogs into East Asia can be ascertained. One southward into eastern China and the other southwestward to western China, gradually forming the AEC and the AWC groups (Fig. 3a) during 8 to 5 ka BP. The emergence of haplogroups A1a, B, and C in East Asia indicated a maternal genetic contribution from western to eastern Eurasian dogs approximately 4 ka BP, with the western Eurasian influence increasing rapidly after 3 ka BP (Fig. 2c). Subsequently, it largely replaced most primary maternal lineages in northern East Asia during the ensuing periods. We identified at least three major mitogenome sub-haplogroups of haplogroup A (A1a, A1b, and A3) that represent two major dispersal waves onto the Qinghai-Tibet Plateau in ancient times. Further aDNA studies, particularly those incorporating nuclear genome data, from less sampled regions will contribute to filling in the remaining details of the population history and origin of dogs.

## Materials and Methods

### Sample Collection and Age Definition

Samples were obtained from 38 archaeological sites in Gansu (Dabaozishan, *n* = 3; Ganggangwa, *n* = 1; Maojiaping, *n* = 4; Mazongshan, *n* = 1; Shannashuza, *n* = 8; Xichengyi, *n* = 1), Hebei (Xinglong, *n* = 5), Heilongjiang (Harbin, *n* = 7), Jiangsu (Xiaolongtou, *n* = 2), Qinghai (Andaqiha, *n* = 1; Jinchankou, *n* = 5; Majiatai, *n* = 1; Nantan, *n* = 1; Talitaliha, *n* = 1; Yangqu, *n* = 1), Shaanxi (Donglongshan, *n* = 4; Laoniupo, *n* = 1; Quanhucun, *n* = 7; Shenheyuan, *n* = 3; Shimao, *n* = 3; Xinjie, *n* = 10; Yangguanzhai, *n* = 10), Shandong (Beiqian, *n* = 2; Dinggong, *n* = 9; Jiaojia, *n* = 4; Pengjiazhuang, *n* = 1; Shicun, *n* = 1; Tangye, *n* = 4; Zhuguogucheng, *n* = 2), Xinjiang (Qiongkeke, *n* = 1; Jirentai, *n* = 1), Xizang (Butaxiongqu, *n* = 1; Dama, *n* = 1; Meilongdapu, *n* = 1; Qulongsaza, *n* = 1; Sangdalongguo, *n* = 5), and Zhejiang (Jingtoushan, *n* = 5; Tianluoshan, *n* = 1) provinces of China (Fig. 1a; see supplementary table S1, Supplementary Material online).

Twenty-nine specimens were selected for accelerator mass spectrometry radiocarbon dating, which regards the average value of the calibrated age range as the final age (see supplementary table S1, Supplementary Material online). Eleven specimens had radiocarbon age results from Zhang et al. (2020). Individuals from Harbin, Heilongjiang were excavated from the river bottom, without any cultural information. To estimate the age of samples from Harbin, Heilongjiang (including one selected for radiocarbon dating), we performed a Bayesian reconstruction of the phylogenetic tree using BEAST v1.10.4 (Suchard et al. 2018), calibrating the molecular clock using tip ages for all ancient samples with a finite radiocarbon date (see supplementary fig. S6 and table S4, Supplementary Material online). We assumed a strict molecular clock with a substitution rate of 8.1941 × 10^−8^ ± 9.656 × 10^−9^ substitutions per site per year and other parameters were from Zhang et al. (2020), except a Bayesian skygrid coalescent model to account for the complex cross-generic demographic history of the included taxa (see supplementary fig. S6 and table S4, Supplementary Material online). Four individuals from Harbin, Heilongjiang gave dates ranging from 16.1 to 6.3 ka BP (see supplementary fig. S6 and table S4, Supplementary Material online). A high-quality sequence belonging to haplogroup A1a was estimated to be approximately 10.2 ka BP (95% HPD interval, 13.5 to 6.8 ka BP) old, and represented the deepest lineage of haplogroup A1a (see supplementary fig. S6 and table S4, Supplementary Material online). The archaeological age information was used for the ages of the remaining 74 samples (see supplementary table S1, Supplementary Material online).

We prioritized East Asia and chose diverse representative sequences from Europe, America, Oceania, and Africa. For computational efficiency, we carefully selected representative sequences for analysis from homogeneous sequences in each region. Finally, we selected 144 sequences of ancient and modern dogs belonging to the main dog haplogroup (haplogroup A) from previous publications (see supplementary table S2, Supplementary Material online).

### Definition of Geographic Regions

We defined the geographic regions utilized in our study as follows: Ancient northern dogs: referring to dog populations from northeast Eurasia and northern East Asia; Northern/southern East Asia: the division between northern and southern East Asia is demarcated by the Qinling-Huaihe line, extending from the Huaihe River to the Qinling mountains (see Fig. 1b and supplementary fig. S2, Supplementary Material online); Northeastern Eurasia: encompassing eastern Siberia and the northern region of northern East Asia (e.g. Harbin); Eastern Eurasia: comprising northern East Asia and eastern Siberia; Western Eurasia: comprising Europe and the Middle East.

### DNA Extraction, Amplification, and Sequencing

Bone powder (13.8 to 145.1 mg) was prepared from each specimen using a Dremel tool and single-use drill bits. Ancient DNA laboratory work following the same protocol described in Zhang et al. (2020) was conducted at the Institute of Vertebrate Paleontology and Paleoanthropology, Chinese Academy of Sciences in Beijing, China. Double-stranded or single-stranded libraries with no uracil-DNA-glycosylase (UDG), half UDG, or full UDG treatment were prepared from 102 new samples (see supplementary table S1, Supplementary Material online). We utilized the probes for 242 mammalian mtDNAs to enrich for mammalian and human mtDNA (Slon et al. 2016). Sequencing for libraries followed Zhang et al. (2020).

### Read Processing and Authenticity Criteria for aDNA

During DNA extractions and in all polymerase chain reaction (PCR) reactions, negative controls were used for every tenth sample. All reagents of molecular biology grade were used, and decontamination using bleach and/or ultraviolet irradiation was used for all working surfaces and equipment. Only samples that were consistent with repeated extractions and amplifications were included in the analyses. Overlapping mate-pairs were merged using the program leeHom (https://github.com/grenaud/leeHom) (Renaud et al. 2014) with the parameter “-ancientdna”, and the adapter sequences were trimmed. Merged and trimmed reads were mapped using Burrows-Wheeler Alignment tool (BWA v.0.7.17) (Li and Durbin 2010) (bwa bam2bam -g Reference Input.bam) to the dog mitochondrial reference (GenBank Accession Number: NC_002008) (Kim et al. 1998). Duplicates were then identified and removed by bamrmdup v.0.2 (https://github.com/mpieva/biohazard-tools). Reads having at least 30 bp in length and minimum mapping quality score of 30 were kept for analysis. All samples showed high C > T frequencies at the 5′ end of fragments, consistent with aDNA damage patterns. FASTA sequences were generated from bam files using a script from https://github.com/mpieva/mapping-iterative-assembler (mia -m Output -U -C -k12 -c -H1). As a result, we obtained a mean 102.88-fold (range: 5.28 to 843.06) coverage for the 102 newly sequenced complete mitochondrial genomes (see supplementary table S1, Supplementary Material online). Present-day contamination was estimated through a likelihood-based method by comparing sequenced mitochondrial genomes with those found in 626 present-day dogs and wolves worldwide, whose mtDNA haplotypes were treated as the contaminating population (Fu et al. 2013). The estimated contamination rate for all samples was less than 5% (see supplementary table S1, Supplementary Material online). To address potential reference bias, we utilize a gray wolf (GenBank Accession Number: NC_008092) mitochondrial genome as a reference to remap all individual sequences from this study. Reanalysis was conducted for all newly generated sequences by comparing the outcomes using both the dog and gray wolf mitochondrial references. The results demonstrated that our analyses were not influenced by reference bias. New sequences obtained in this study have been deposited in GenBank under accession numbers: PP454580 - PP454699.

### Haplogroup Nomenclature

Nearly all modern dogs worldwide fall into one of four monophyletic haplogroups (A, B, C, or D), with the majority belonging to haplogroup A (Pang et al. 2009; Thalmann et al. 2013; Peng et al. 2015; Song et al. 2016; Ameen et al. 2019; Zhang et al. 2020; Perri et al. 2021). Although the four monophyletic haplogroups (A, B, C, and D) matched well in different studies, the sub-haplogroups in haplogroup A were unclear. No consensus study has emerged that clarified this iusse satisfactorily. The more than 100 ancient East Asian dog mitogenomes obtained in this study provided an opportunity to revise the mitogenome haplogroup nomenclature system based on comparison with different references (see supplementary tables S1 and S3, Supplementary Material online) (Thalmann et al. 2013; Peng et al. 2015; Song et al. 2016; Ameen et al. 2019; Zhang et al. 2020; Perri et al. 2021).

We compared the haplogroup nomenclature of six influential studies with our study and made a comprehensive comparison table based on the results of phylogenetic analyses. We also wanted a nomenclature representative of the relationships among sub-haplogroups (see supplementary table S3, Supplementary Material online). We employed ***mitotoolpy-seq.py*** (http://dometree.kiz.ac.cn/) to conduct the major haplogroups classification (Peng et al. 2015), and checked the results based on supplementary table S3, Supplementary Material online. We have confirmed the consistency of major haplogroups (e.g. haplogroups A, B, C, and D) across all the studies (Thalmann et al. 2013; Peng et al. 2015; Song et al. 2016; Ameen et al. 2019; Zhang et al. 2020; Perri et al. 2021). In this study, we followed the nomenclature of Zhang et al. (2020), Ameen et al. (2019), and Perri et al. (2021). Four sub-haplogroups of A1b were the same as Zhang et al. (2020), and we changed A3 (Peng et al. 2015; Song et al. 2016; Zhang et al. 2020) to A1c, A5 (Peng et al. 2015; Song et al. 2016; Zhang et al. 2020) to A2c, A6 (Peng et al. 2015; Song et al. 2016; Zhang et al. 2020) to A3, and Unassigned A (Zhang et al. 2020) to A4 (see supplementary table S3, Supplementary Material online).

### Haplogroup Proportions in Different Areas and Time Periods

We selected modern and ancient data from Zhang et al. (2020) and Perri et al. (2021), combined with high-quality sequences from our dataset, to draw the haplogroup distribution map using Arcgic v.10.8 (www.esri.com). We compared the distribution of haplogroups A, B, and C and sub-haplogroup A from East Asia, Europe, Siberia, North America, Australia, and Pacific Islands (Fig. 1b; see supplementary fig. S2, Supplementary Material online). To further investigate the changes in haplogroup A proportion in East Asia over different time periods (five time periods spanning from the Palaeolithic to historical periods in ancient individuals: >5 ka BP; 5 to 4 ka BP; 4 to 3 ka BP; 3 to 2 ka BP; <2 ka BP). A total of 119 ancient individuals and 121 modern individuals we used after combining with the data collected by Zhang et al. (2020) and Cai et al. (2023), visualized in RStudio (https://www.rstudio.com/). Modern individuals from Anhui, Guangdong, Guangxi, Guizhou, Hainan, Hunan, Jiangxi, Sichuan, and Yunnan provinces were assigned as southern East Asian dogs, and individuals from Henan, Liaoning, Gansu, Hebei, Qinghai, Shaanxi, Shanxi, Shandong provinces were classified as northern East Asian dogs.

### Network Construction

To investigate the genetic population structure of haplogroup A in the dog populations, we selected ancient (*n* = 69) and modern (*n* = 76) sequences (from Asia, Africa, America, Europe, and Oceania), combined with high-quality sequences from our dataset (*n* = 106, see supplementary table S1, Supplementary Material online). The median-joining network of each sub-haplogroups was constructed in PopART v.1.7.1 (Leigh and Bryant 2015). Sequences with more than 1,000 “N” sites and several modern sequences were deleted. Finally, we used 202 sequences for an overview of haplogroup A (see supplementary fig. S5A, Supplementary Material online). Fifty-five sequences belonging to haplogroup A1a were selected for network construction (see supplementary fig. S5B, Supplementary Material online). After deleting sequences with more than 1,000 “N” sites (excluding the Tianluoshan individual from Zhejiang), we selected 82 sequences belonging to haplogroup A1b for network construction (Fig. 2b). Network construction of haplogroups A2 and A3 used 57 and 19 individuals, respectively (Fig. 2d; see supplementary fig. S5C and tables S1 and S2, Supplementary Material online).

### Phylogenetic Trees

Although missing sites in aDNA sequences are randomly distributed and generally have minimal impact on the topology of your phylogenetic trees (Hovmöller et al. 2013; Thalmann et al. 2013; Zhang et al. 2020). To prevent potential influence by the missing data, we exclusively utilized sequences with missing values below 15% (see supplementary table S1, Supplementary Material online). A dataset with all existing sub-haplogroups of haplogroup A of both ancient and modern dog mitochondrial genomes was built, resulting in a final dataset of 188 individuals (one wolf as outgroup) for Bayesian phylogenetic analysis. Bayesian phylogenetic tree built by BEAST v.1.10.4 (Suchard et al. 2018), with the same setting of Zhang et al. (2020) (sequence length is 16,039 bp, and the substitution rate is 8.1941 × 10^−8^ ± 9.656 × 10^−9^ substitutions per site per year). The TrN+I+G substitution model was chosen by JModelTest2 v.2.1.10 (Darriba et al. 2012). The strict molecular clock and Bayesian skyline with a piecewise-linear tree model was selected following Zhang et al. (2020). A total of 50 million iterations, sampling at every 5,000 iterations and ensuring that all effective sample size values were greater than 300 in Tracer v1.6.0 (Rambaut et al. 2018). The phylogenetic tree was visualized in FigTree v.1.4.0, with 20% of the trees discarded as burn-in (Fig. 1c; see supplementary fig. S3, Supplementary Material online). The Ne_f_ was estimated based on the same dataset and parameter used, utilizing the Bayesian skyline plot (Fig. 2a).

We used 258 haplogroup A sequences to construct the maximum likelihood phylogenetic tree by RAxML (Stamatakis 2014) using a General Time Reversible model with a GTR+G+I model and setting the bootstrap value to 1,000 times. The phylogenetic tree clearly grouped all sequences that covered as many lineages as possible and represented almost all mitochondrial haplogroups. A consensus tree was calculated by applying *phylip consense*, using 1,000 candidate trees we produced, and was visualized in FigTree v.1.4.0 (see supplementary fig. S4, Supplementary Material online).

## Supplementary Material

msae062_Supplementary_Data

## Data Availability

New sequences obtained in this study have been archived in GenBank under accession numbers: PP454580 - PP454699. All software are cited in the Materials and methods and are publicly available.
