# Supplementary material for: Ancient Mitogenomes Reveal the Maternal Genetic History of East Asian Dogs
Source: Mol Biol Evol. 2024 Mar 20;41(4):msae062. doi: 10.1093/molbev/msae062 (PMC11003542; doi:10.1093/molbev/msae062)
Supplement: msae062_Supplementary_Data [file msae062_supplementary_data.zip › supplementary_table_S3._Haplogroup_comparison.pdf]

supplementary table S3. Haplogroup nomenclature comparison of six influential studies with our study. References used in this table, I: Thalmann et al. 2013; II: Peng et al. 2015; III: Song et al. 2016; IV: Ameen et al. 2019; V: Zhang et al. 2020; VI: Perri et al. 2021.

Haplogroup	I	II, and III	IV	V	VI	This study		Description
A	A	A1	A1a	A1a	-	A1a		The most common haplogroup among modern dogs, especially European breed dogs. The deepest lineage of this sub-haplogroup is from Harbin, Heilongjiang. East Asian dogs within this sub-haplogroup increasing sharply after about 3 ka BP (Fig. 2C).
		A2	A1b	A1b	A1b	A1b	A1b1	Typical sub-haplogroup in "Ancient Eastern China" (AEC, Shandong and Zhejiang), as the dogs from eastern China with deeper ancestry and older records (Shandong, ~5-3 ka BP) than western China (Shaanxi, ~2 ka BP and Qinghai, ~1.6 ka BP).
							A1b2	Ancient dogs from East Asia, commonly found in both AEC and "Ancient Western China" (AWC, Shaanxi, Gansu, Qinghai, and Xizang), and ancient dogs from pre-colonial Pacific Islands.
							A1b3	Typical sub-haplogroup in AWC, as the dogs form western China with deeper ancestry and older records (Gansu, ~5-3 ka BP; Shaanxi, ~5-3 ka BP; and Qinghai, ~4 ka BP) than eastern China (Shandong, ~2 ka BP).
							A1b4	One branch of this sub-haplogroup found in modern Australian dingoes and New Guinea Singing dogs, the other branch found in ancient and modern dogs from northeastern Siberia (e.g. ~1.8 ka BP, western Chukotka) and North America.
		A3	-	A3	-	A1c		Dogs occurred in both AEC and AWC, also found in a few modern dogs from southern East Asia and Southeast Asia.
		A4	A2a	A2a	A2a	A2a		Mainly found in Innit dogs, also found in ancient dogs from eastern Eurasia (e.g. China: Heilongjiang and Shandong; Russia: Far East and Baikal).
			A2b	A2b	A2b	A2b		Mainly found in ancient dogs from Siberia and pre-contact dogs in North America.
		A5	-	A5	-	A2c		This sub-haplogroup at the basal of A2a and A2b. Dogs within sub-haplogroup A2c widely distributed in ancient East Asia (e.g. Zhejiang, Shaanxi, Gansu, and Shandong) and even Pacific island (e.g. East Timor). However, dogs within this sub-haplogroup only found in southern China in present time (e.g. Guizhou, Yunnan, Jiangxi, and Hainan).
		A6	-	A6	-	A3		This sub-haplogroup widely distributed in ancient East Asia (e.g. Hebei, Shaanxi, Gansu, Qinghai, and Xizang) and Siberia (e.g. New Siberian Islands), but only found in modern dogs from the Qinghai-Tibet Plateau and surrounding area (Fig. 2D).

		-	-	Unassigned A	-	A4	One individual from Lacha, Russia (from western Eurasia, ~11 ka BP) basal to all other lineages (from western China, ~5 ka BP).
B	B	B	-	-	-	B	Haplogroup B might introgressed from wolves (Thalmann et al., 2013), and found in Xinjiang (Qiongkeke, ~2.9 ka BP), Shandong (Pengjiazhuang, ~3.5 ka BP; and Tangye, ~3 ka BP).
C	C	C	-	C	C	C	Haplogroup C is an typical western Eurasian-like type (Frantz et al. 2016; Ollivier et al. 2018). Two individuals from Jinchankou, Qinghai (~4 ka BP) indicated the genetic connection at least date back to 4 ka BP.
D	D	D	-	-	-	-	No individual belong to haplogroup D in this study. Haplogroup D shared with dogs and wolves (Peng et al. 2015).
