# Supplementary figures and images for: Ancient Mitogenomes Reveal the Maternal Genetic History of East Asian Dogs
Source: Mol Biol Evol. 2024 Mar 20;41(4):msae062. doi: 10.1093/molbev/msae062 (PMC11003542; doi:10.1093/molbev/msae062)

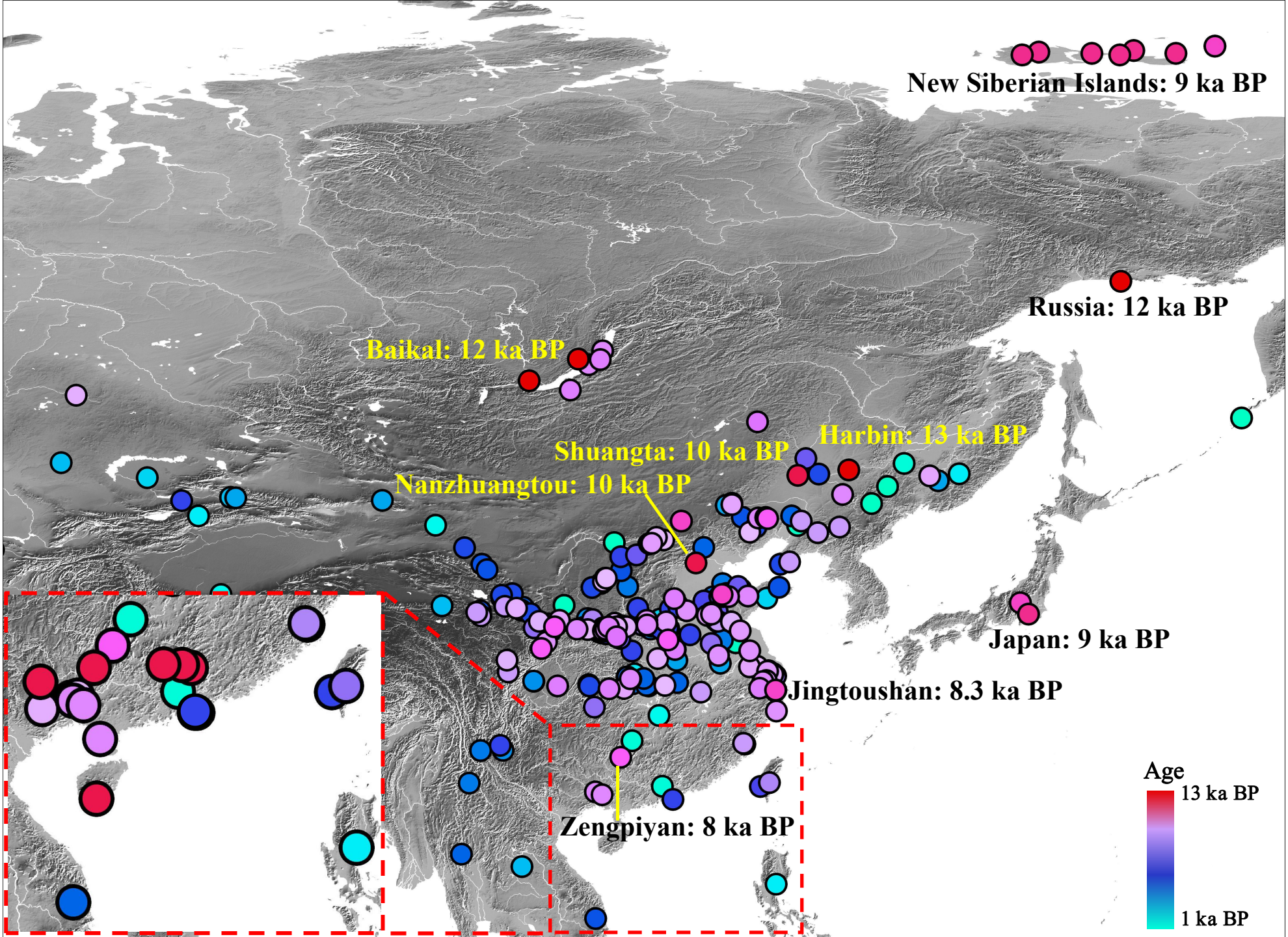

Supplement: msae062_Supplementary_Data [file msae062_supplementary_data.zip › supplementary fig. S1.pdf]

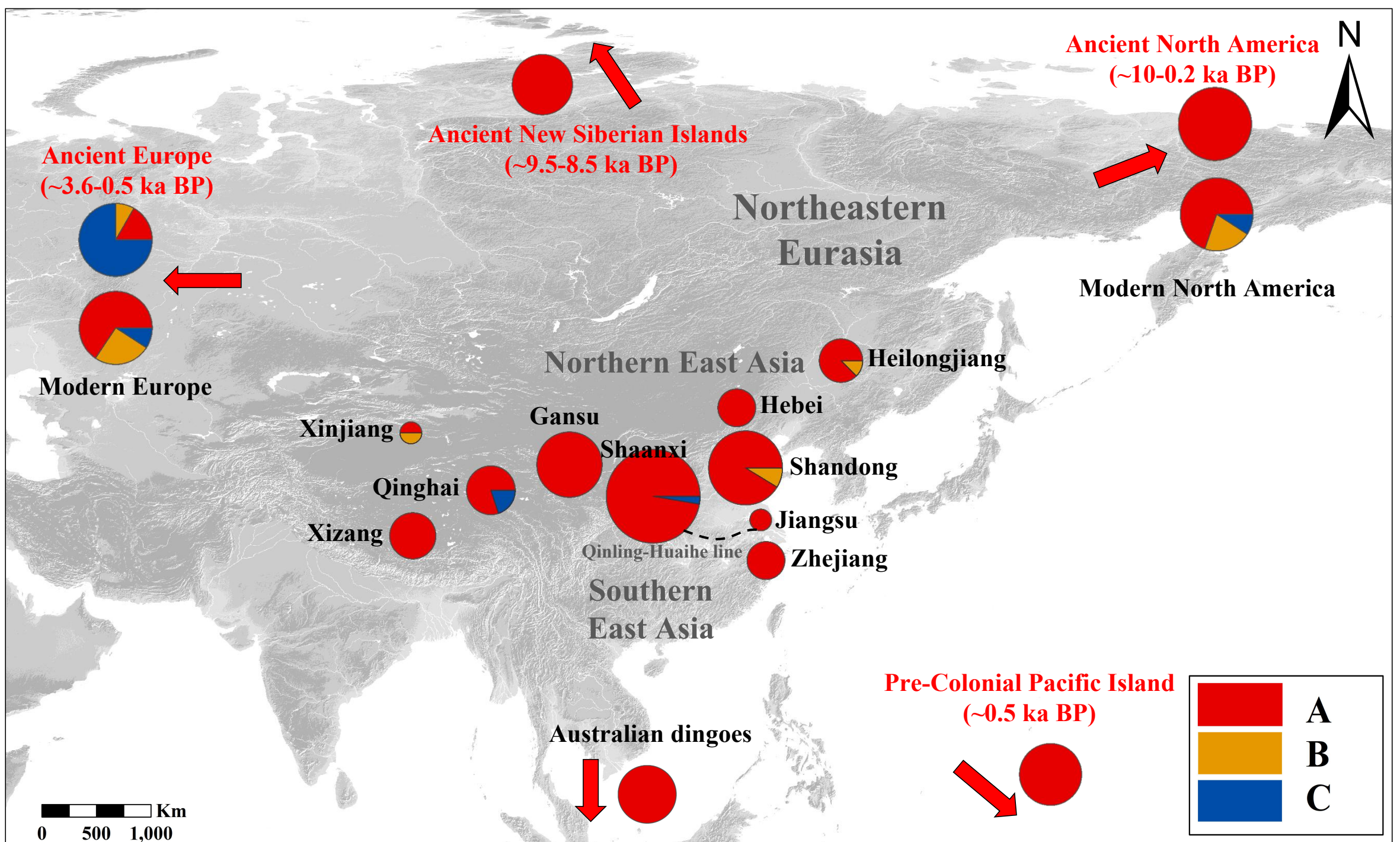

Supplement: msae062_Supplementary_Data [file msae062_supplementary_data.zip › supplementary fig. S2.pdf]

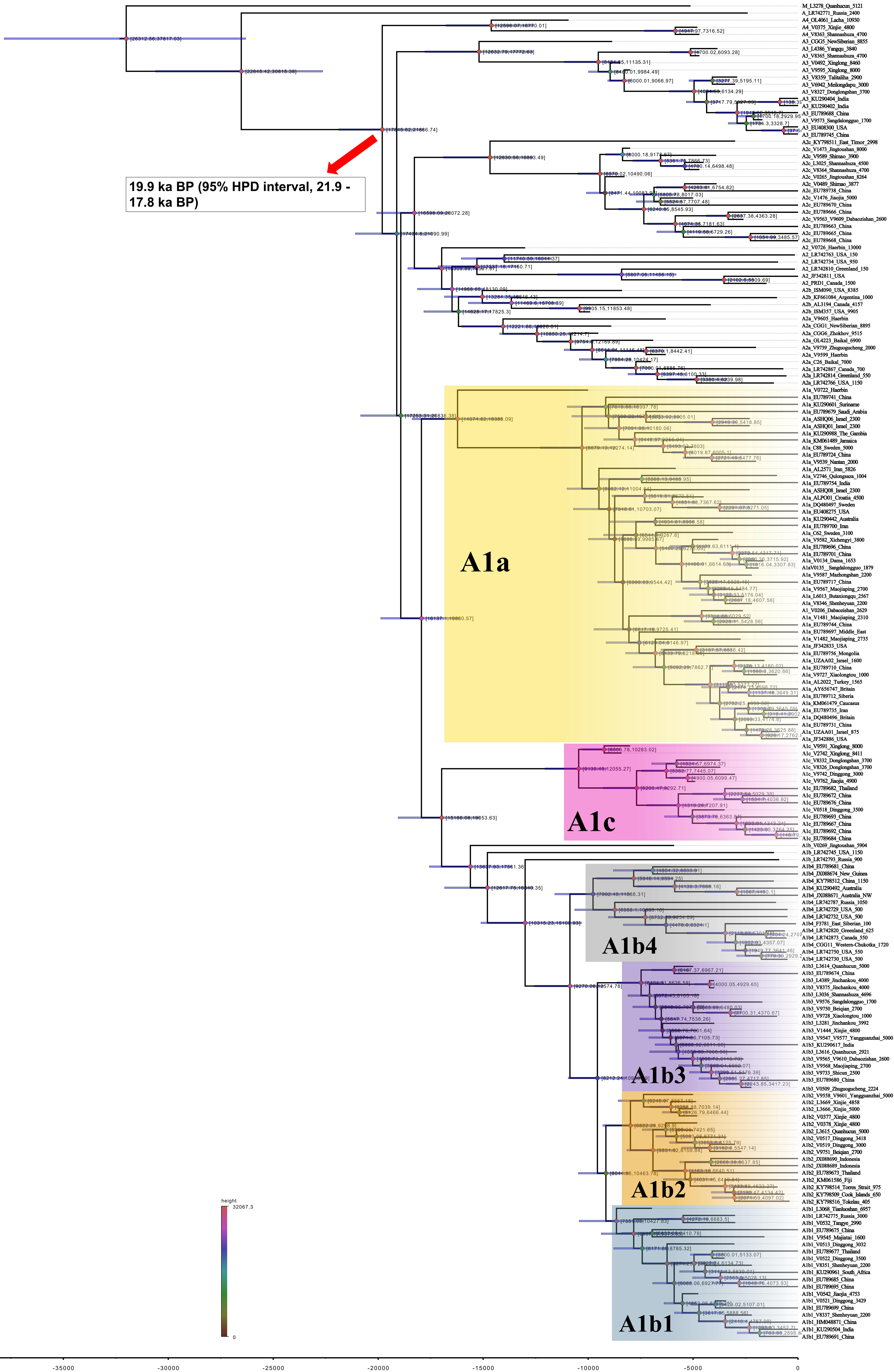

Supplement: msae062_Supplementary_Data [file msae062_supplementary_data.zip › supplementary fig. S3.pdf]

A

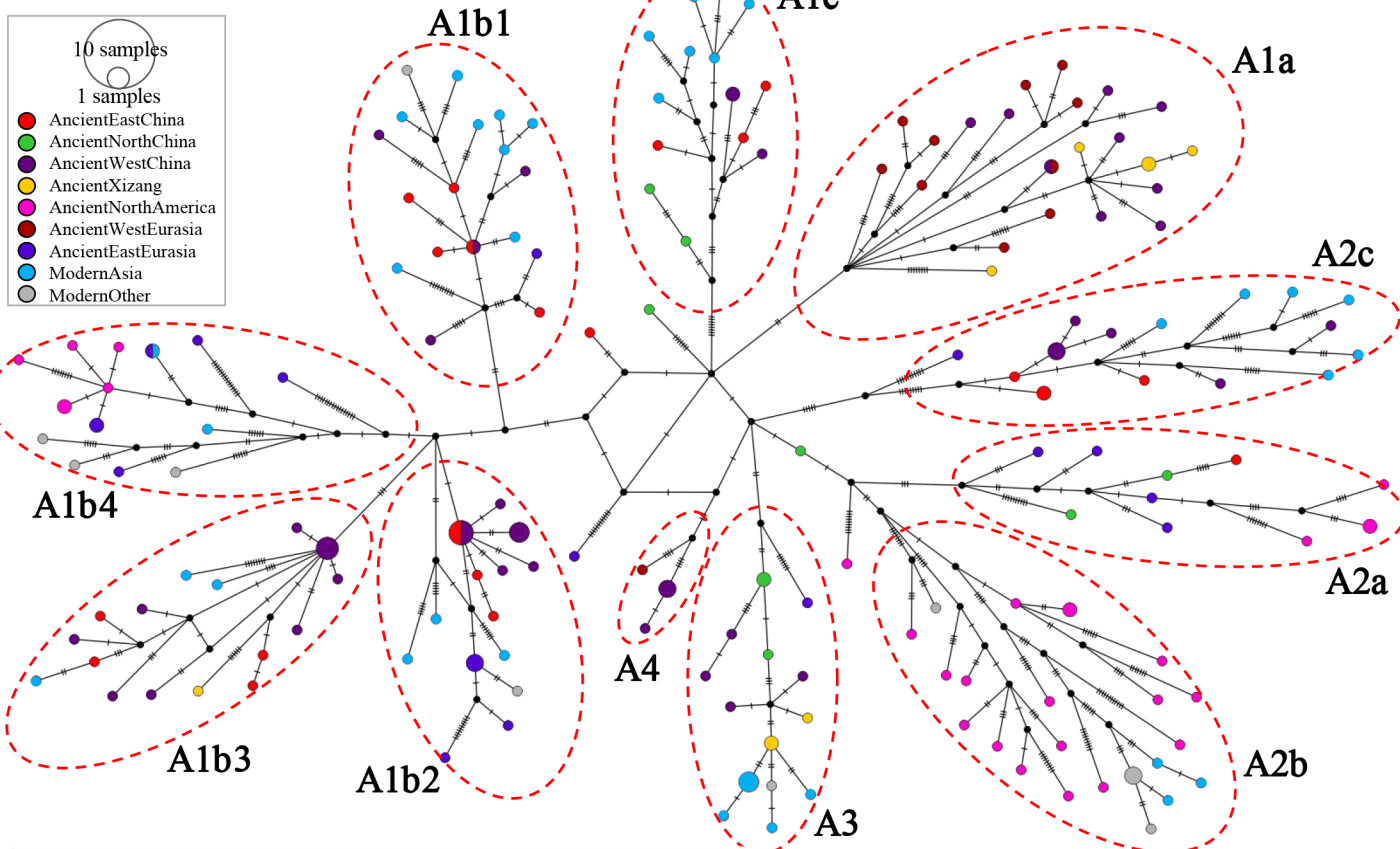

B

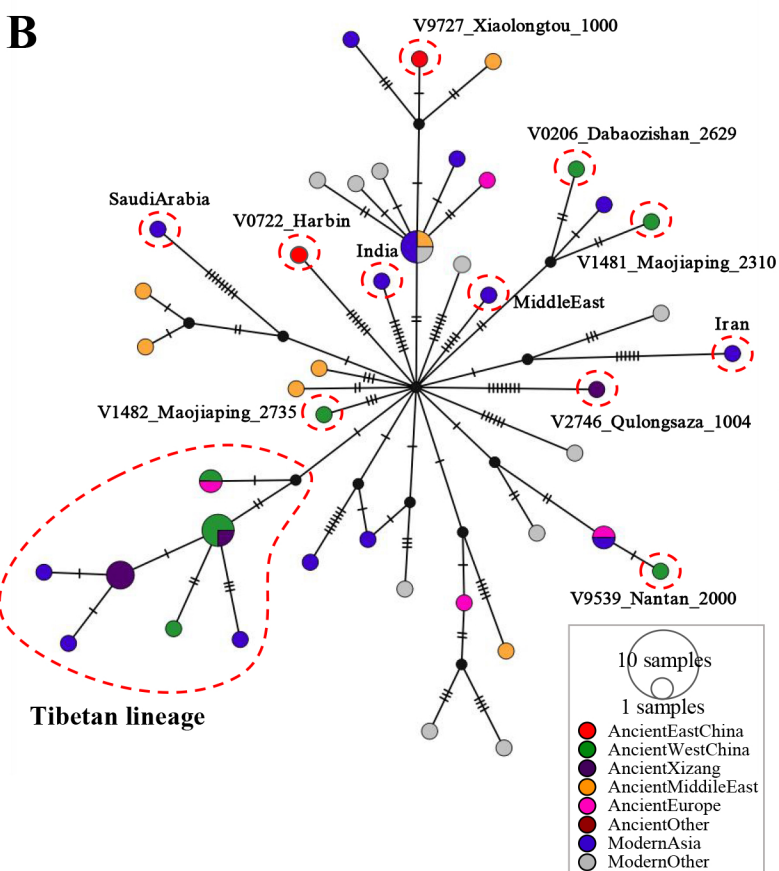

C

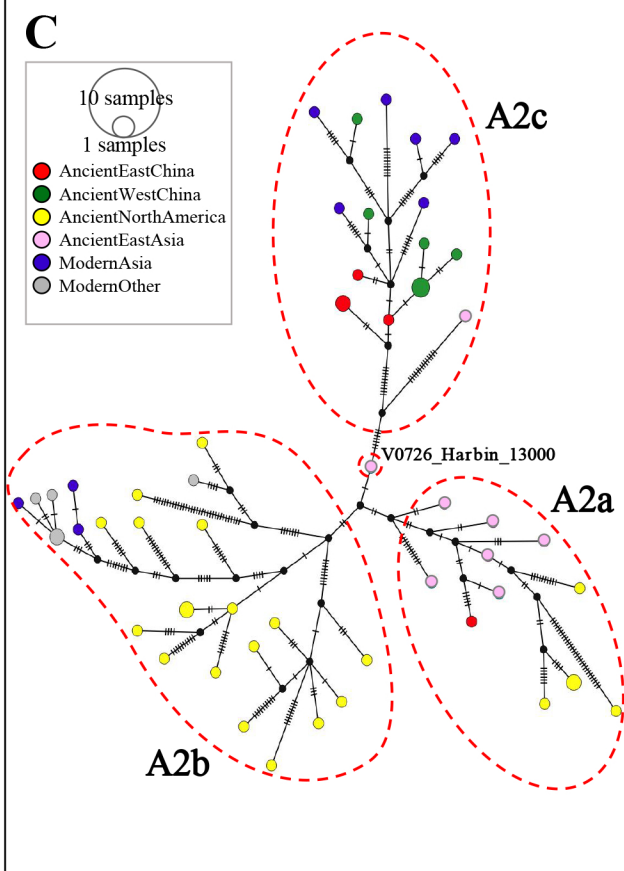

Supplement: msae062_Supplementary_Data [file msae062_supplementary_data.zip › supplementary fig. S5.pdf]

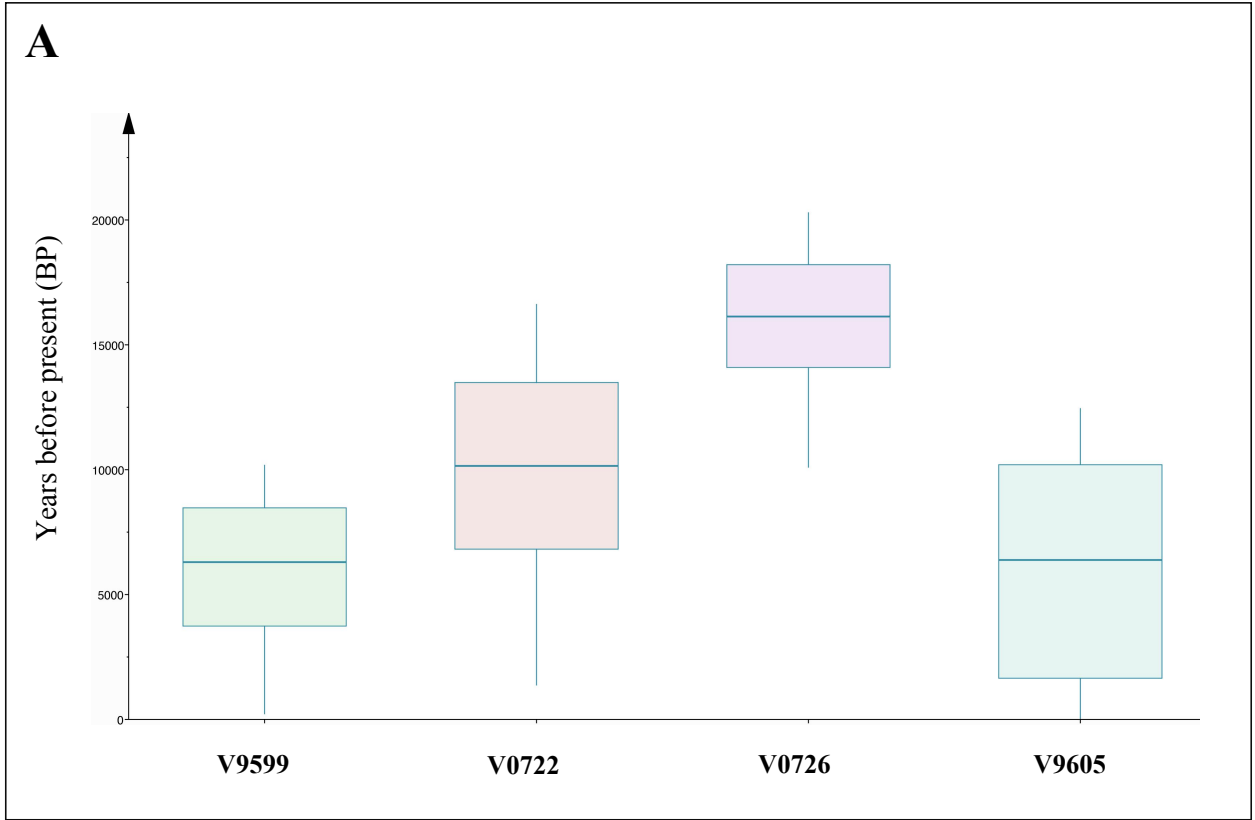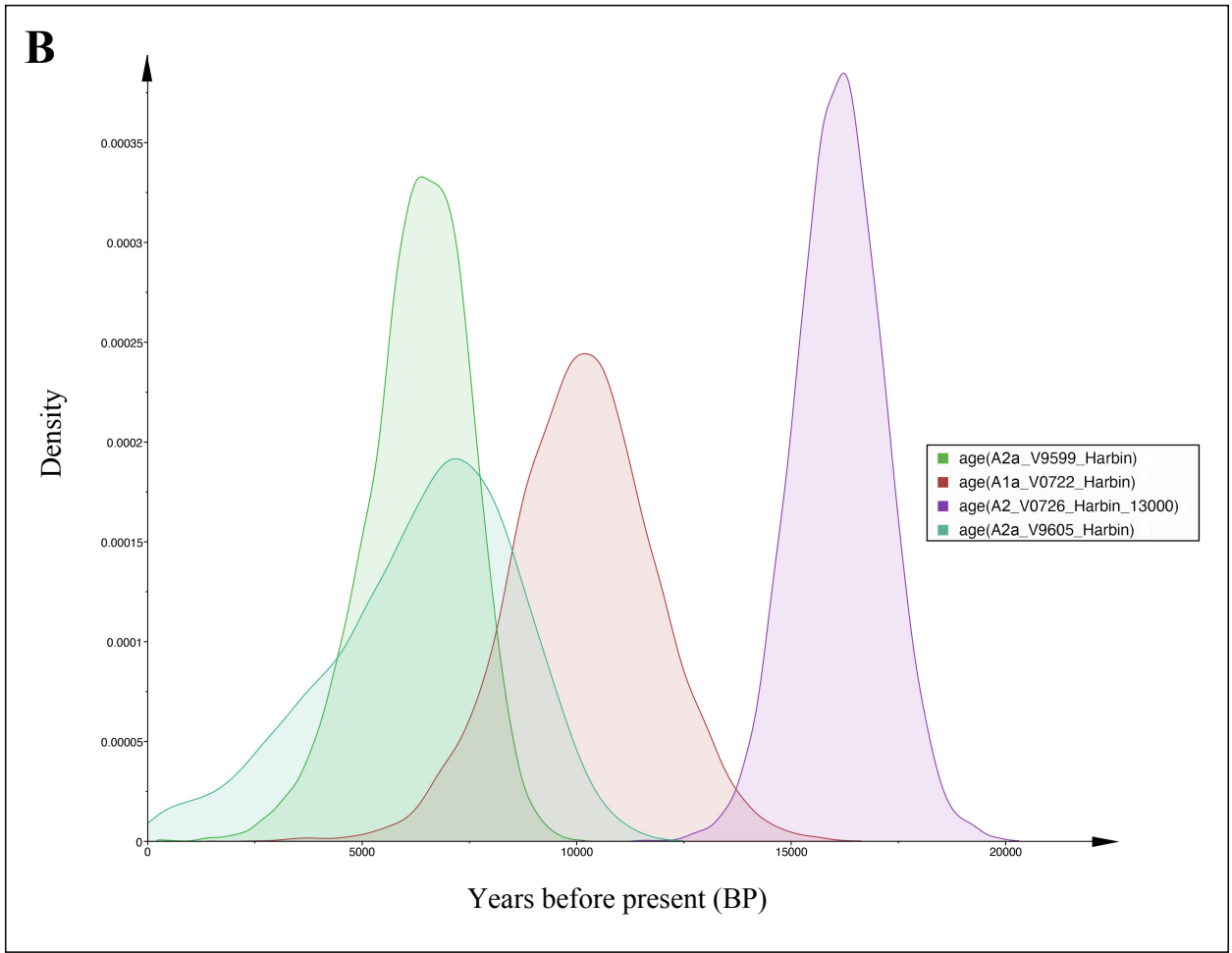

Supplement: msae062_Supplementary_Data [file msae062_supplementary_data.zip › supplementary fig. S6.pdf]
