# Supplementary material for: Ancient Mitogenomes Reveal the Maternal Genetic History of East Asian Dogs
Source: Mol Biol Evol. 2024 Mar 20;41(4):msae062. doi: 10.1093/molbev/msae062 (PMC11003542; doi:10.1093/molbev/msae062)
Supplement: msae062_Supplementary_Data [file supplementary fig. S4.pdf › supplementary fig. S4.pdf]

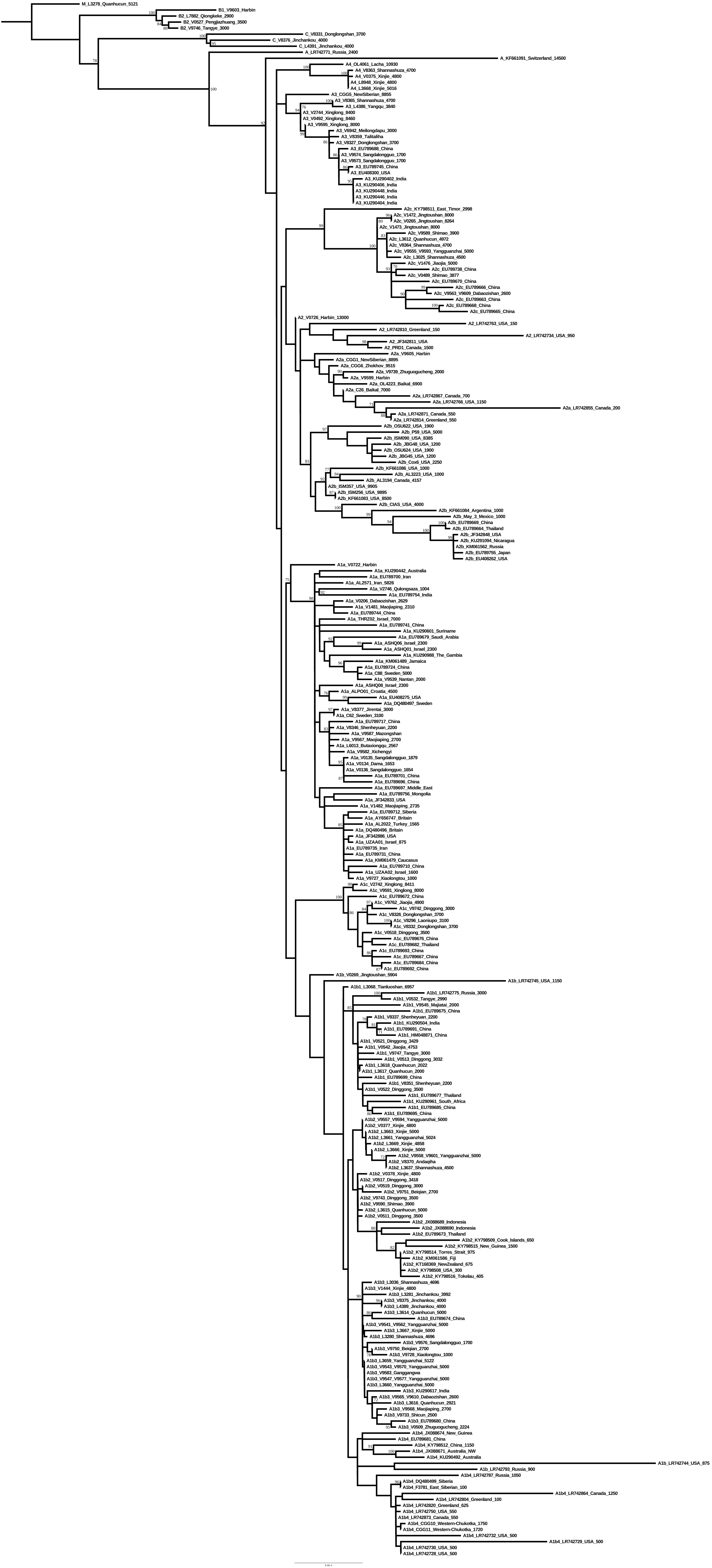
M_L3278_Quanhucun_5121
B1_V9603_Harbin
B2_L7882_Qiongkeke_2900
B2_V0527_Pengjiazhuang_3500
B2_V9746_Tangye_3000
C_V8331_Donglongshan_3700
C_V8376_Jinchankou_4000
C_L4391_Jinchankou_4000
A_LR742771_Russia_2400
A_KF661091_Switzerland_14500
A4_OL4061_Lacha_10930
A4_V8363_Shannashuza_4700
A4_V0375_Xinjie_4800
A4_L8948_Xinjie_4800
A4_L3668_Xinjie_5016
A3_CGG5_NewSiberian_8855
A3_V8365_Shannashuza_4700
A3_L4386_Yangqu_3840
A3_V2744_Xinglong_8400
A3_V0492_Xinglong_8460
A3_V9595_Xinglong_8000
A3_V6942_Meilongdapu_3000
A3_V8359_Talitaliha
A3_V8327_Donglongshan_3700
A3_EU789688_China
A3_V9574_Sangdalongguo_1700
A3_V9573_Sangdalongguo_1700
A3_EU789745_China
A3_EU408300_USA
A3_KU290402_India
A3_KU290406_India
A3_KU290448_India
A3_KU290446_India
A3_KU290404_India
A2c_KY798511_East_Timor_2998
A2c_V1472_Jingtoushan_8000
A2c_V0265_Jingtoushan_8264
A2c_V1473_Jingtoushan_8000
A2c_V9589_Shimao_3900
A2c_L3612_Quanhucun_4972
A2c_V8364_Shannashuza_4700
A2c_V9555_V9593_Yangguanzhai_5000
A2c_L3025_Shannashuza_4500
A2c_V1476_Jiaojia_5000
A2c_EU789738_China
A2c_V0489_Shimao_3877
A2c_EU789670_China
A2c_EU789666_China
A2c_V9563_V9609_Dabaozishan_2600
A2c_EU789663_China
A2c_EU789668_China
A2c_EU789665_China
A2_V0726_Harbin_13000
A2_LR742763_USA_150
A2_LR742810_Greenland_150
A2_LR742734_USA_950
A2_JF342811_USA
A2_PRD1_Canada_1500
A2a_V9605_Harbin
A2a_CGG1_NewSiberian_8895
A2a_CGG6_Zhokhov_9515
A2a_V9739_Zhuguogucheng_2000
A2a_V9599_Harbin
A2a_OL4223_Baikal_6900
A2a_C26_Baikal_7000
A2a_LR742867_Canada_700
A2a_LR742766_USA_1150
A2a_LR742855_Canada_200
A2a_LR742871_Canada_550
A2a_LR742814_Greenland_550
A2b_OSU622_USA_1900
A2b_P59_USA_5000
A2b_ISM090_USA_8385
A2b_JBG48_USA_1200
A2b_OSU624_USA_1900
A2b_JBG45_USA_1200
A2b_Cox6_USA_2250
A2b_KF661086_USA_1000
A2b_AL3223_USA_1000
A2b_AL3194_Canada_4157
A2b_ISM357_USA_9905
A2b_ISM256_USA_9895
A2b_KF661083_USA_8500
A2b_CIAS_USA_4000
A2b_KF661084_Argentina_1000
A2b_May_3_Mexico_1000
A2b_EU789669_China
A2b_EU789664_Thailand
A2b_JF342848_USA
A2b_KU291094_Nicaragua
A2b_KM061562_Russia
A2b_EU789755_Japan
A2b_EU408262_USA
A1a_V0722_Harbin
A1a_KU290442_Australia
A1a_EU789700_Iran
A1a_AL2571_Iran_5826
A1a_V2746_Qulongsaza_1004
A1a_EU789754_India
A1a_V0206_Dabaozishan_2629
A1a_V1481_Maojiaping_2310
A1a_EU789744_China
A1a_THRZ02_Israel_7000
A1a_EU789741_China
A1a_KU290601_Suriname
A1a_EU789679_Saudi_Arabia
A1a_ASHQ06_Israel_2300
A1a_ASHQ01_Israel_2300
A1a_KU290988_The_Gambia
A1a_KM061489_Jamaica
A1a_EU789724_China
A1a_C88_Sweden_5000
A1a_V9539_Nantan_2000
A1a_ASHQ08_Israel_2300
A1a_ALPO01_Croatia_4500
A1a_EU408275_USA
A1a_DQ480497_Sweden
A1a_V8377_Jirentai_3000
A1a_C62_Sweden_3100
A1a_EU789717_China
A1a_V8346_Shenheyuan_2200
A1a_V9587_Mazongshan
A1a_V9567_Maojiaping_2700
A1a_L6013_Butaxiongqu_2567
A1a_V9582_Xichengyi
A1a_V0135_Sangdalongguo_1879
A1a_V0134_Dama_1653
A1a_V0136_Sangdalongguo_1654
A1a_EU789701_China
A1a_EU789696_China
A1a_EU789697_Middle_East
A1a_EU789756_Mongolia
A1a_JF342833_USA
A1a_V1482_Maojiaping_2735
A1a_EU789712_Siberia
A1a_AY656747_Britain
A1a_AL2022_Turkey_1565
A1a_DQ480496_Britain
A1a_JF342886_USA
A1a_UZAA01_Israel_875
A1a_EU789735_Iran
A1a_EU789731_China
A1a_KM061479_Caucasus
A1a_EU789710_China
A1a_UZAA02_Israel_1600
A1a_V9727_Xiaolongtou_1000
A1c_V2742_Xinglong_8411
A1c_V9591_Xinglong_8000
A1c_EU789672_China
A1c_V9762_Jiaojia_4900
A1c_V9742_Dinggong_3000
A1c_V8326_Donglongshan_3700
A1c_V8296_Laoniupo_3100
A1c_V8332_Donglongshan_3700
A1c_V0518_Dinggong_3500
A1c_EU789676_China
A1c_EU789682_Thailand
A1c_EU789693_China
A1c_EU789667_China
A1c_EU789684_China
A1c_EU789692_China
A1b_V0269_Jingtoushan_5904
A1b_LR742745_USA_1150
A1b1_L3068_Tianluoshan_6957
A1b1_LR742775_Russia_3000
A1b1_V0532_Tangye_2990
A1b1_V9545_Majiatai_2000
A1b1_EU789675_China
A1b1_V8337_Shenheyuan_2200
A1b1_KU290504_India
A1b1_EU789691_China
A1b1_HM048871_China
A1b1_V0521_Dinggong_3429
A1b1_V0542_Jiaojia_4753
A1b1_V9747_Tangye_3000
A1b1_V0513_Dinggong_3032
A1b1_L3618_Quanhucun_2022
A1b1_L3617_Quanhucun_2000
A1b1_EU789699_China
A1b1_V8351_Shenheyuan_2200
A1b1_V0522_Dinggong_3500
A1b1_EU789677_Thailand
A1b1_KU290961_South_Africa
A1b1_EU789685_China
A1b1_EU789695_China
A1b2_V9557_V9594_Yangguanzhai_5000
A1b2_V0377_Xinjie_4800
A1b2_L3663_Xinjie_5000
A1b2_L3661_Yangguanzhai_5024
A1b2_L3669_Xinjie_4858
A1b2_L3666_Xinjie_5000
A1b2_V9558_V9601_Yangguanzhai_5000
A1b2_V8370_Andaqiha
A1b2_L3637_Shannashuza_4500
A1b2_V0378_Xinjie_4800
A1b2_V0517_Dinggong_3418
A1b2_V0519_Dinggong_3000
A1b2_V9751_Beiqian_2700
A1b2_V9743_Dinggong_3500
A1b2_V9590_Shimao_3900
A1b2_L3615_Quanhucun_5000
A1b2_V0511_Dinggong_3500
A1b2_JX088689_Indonesia
A1b2_JX088690_Indonesia
A1b2_EU789673_Thailand
A1b2_KY798509_Cook_Islands_650
A1b2_KY798515_New_Guinea_1500
A1b2_KY798514_Torres_Strait_975
A1b2_KM061586_Fiji
A1b2_KT168369_NewZealand_675
A1b2_KY798508_USA_300
A1b2_KY798516_Tokelau_405
A1b3_L3036_Shannashuza_4696
A1b3_V1444_Xinjie_4800
A1b3_L3281_Jinchankou_3992
A1b3_V8375_Jinchankou_4000
A1b3_L4389_Jinchankou_4000
A1b3_L3614_Quanhucun_5000
A1b3_EU789674_China
A1b3_V9541_V9562_Yangguanzhai_5000
A1b3_L3667_Xinjie_5000
A1b3_L3280_Shannashuza_4696
A1b3_V9576_Sangdalongguo_1700
A1b3_V9750_Beiqian_2700
A1b3_V9728_Xiaolongtou_1000
A1b3_L3659_Yangguanzhai_5122
A1b3_V9543_V9570_Yangguanzhai_5000
A1b3_V9583_Ganggangwa
A1b3_V9547_V9577_Yangguanzhai_5000
A1b3_L3660_Yangguanzhai_5000
A1b3_KU290617_India
A1b3_V9565_V9610_Dabaozishan_2600
A1b3_L3616_Quanhucun_2921
A1b3_V9568_Maojiaping_2700
A1b3_V9733_Shicun_2500
A1b3_EU789680_China
A1b3_V0509_Zhuguogucheng_2224
A1b4_JX088674_New_Guinea
A1b4_EU789681_China
A1b4_KY798512_China_1150
A1b4_JX088671_Australia_NW
A1b4_KU290492_Australia
A1b_LR742744_USA_875
A1b_LR742793_Russia_900
A1b4_LR742787_Russia_1050
A1b4_DQ480499_Siberia
A1b4_F3781_East_Siberian_100
A1b4_LR742864_Canada_1250
A1b4_LR742804_Greenland_100
A1b4_LR742820_Greenland_625
A1b4_LR742750_USA_550
A1b4_LR742873_Canada_550
A1b4_CGG10_Western-Chukotka_1750
A1b4_CGG11_Western-Chukotka_1720
A1b4_LR742732_USA_500
A1b4_LR742729_USA_500
A1b4_LR742730_USA_500
A1b4_LR742728_USA_500
